# Analysis of T4SS-induced signaling by *H. pylori* using quantitative phosphoproteomics

**DOI:** 10.3389/fmicb.2014.00356

**Published:** 2014-07-18

**Authors:** Frithjof Glowinski, Carsten Holland, Bernd Thiede, Peter R. Jungblut, Thomas F. Meyer

**Affiliations:** ^1^Department of Molecular Biology, Max Planck Institute for Infection BiologyBerlin, Germany; ^2^The Biotechnology Centre of Oslo, University of OsloOslo, Norway; ^3^Department of Biosciences, University of OsloOslo, Norway

**Keywords:** *H. pylori*, SILAC, phosphoproteomics, CagA, T4SS, tyrosine signaling

## Abstract

*Helicobacter pylori* is a Gram-negative bacterial pathogen colonizing the human stomach. Infection with *H. pylori* causes chronic inflammation of the gastric mucosa and may lead to peptic ulceration and/or gastric cancer. A major virulence determinant of *H. pylori* is the type IV secretion system (T4SS), which is used to inject the virulence factor CagA into the host cell, triggering a wide range of cellular signaling events. Here, we used a phosphoproteomic approach to investigate tyrosine signaling in response to host-pathogen interaction, using stable isotope labeling in cell culture (SILAC) of AGS cells to obtain a differential picture between multiple infection conditions. Cells were infected with wild type *H. pylori* P12, a P12Δ CagA deletion mutant, and a P12Δ PAI deletion mutant to compare signaling changes over time and in the absence of CagA or the T4SS. Tryptic peptides were enriched for tyrosine (Tyr) phosphopeptides and analyzed by nano-LC-Orbitrap MS. In total, 85 different phosphosites were found to be regulated following infection. The majority of phosphosites identified were kinases of the MAPK family. CagA and the T4SS were found to be key regulators of Tyr phosphosites. Our findings indicate that CagA primarily induces activation of ERK1 and integrin-linked factors, whereas the T4SS primarily modulates JNK and p38 activation.

## Introduction

**Figure 1:**
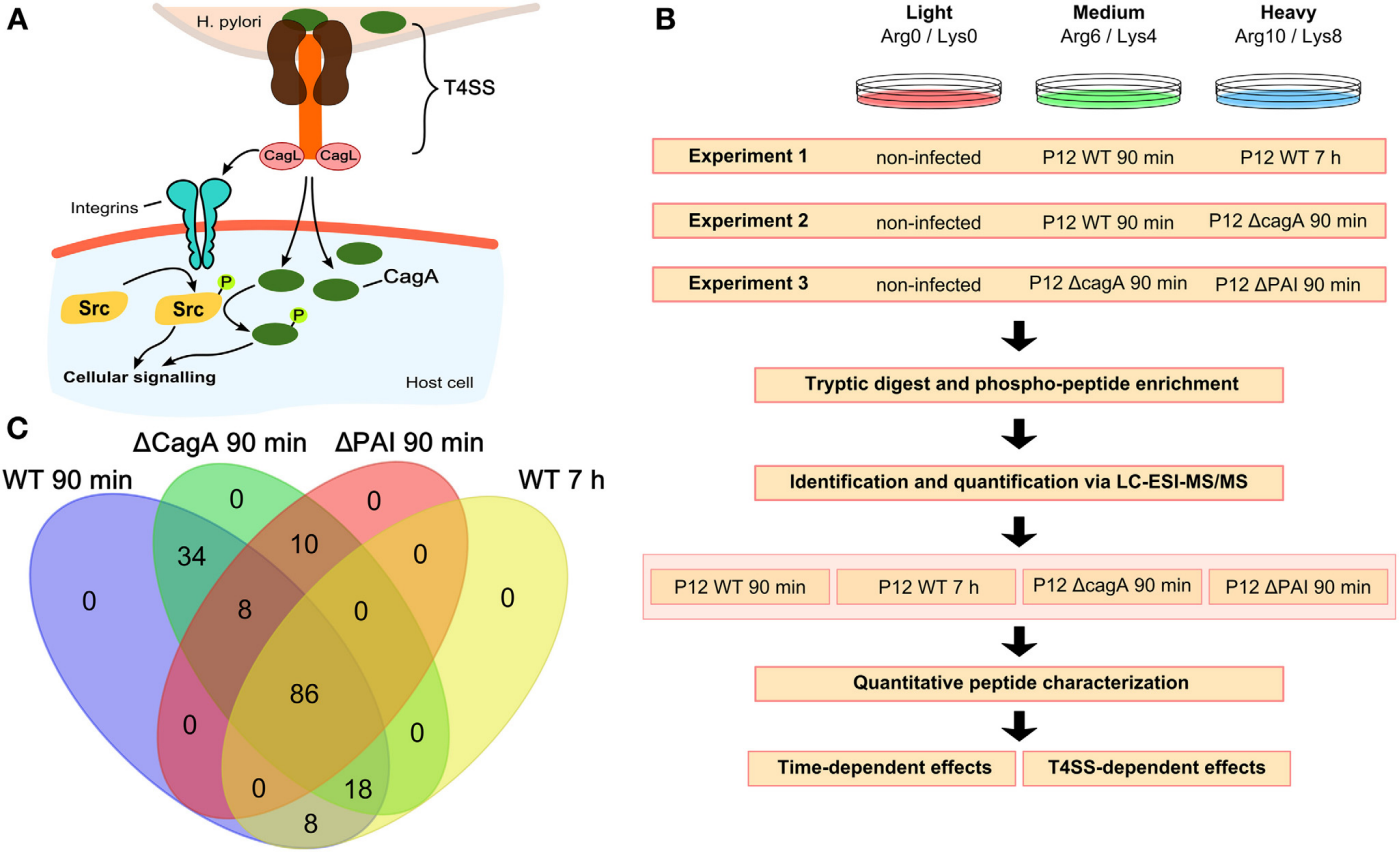
**Experimental setup and analysis workflow. (A)** The *H. pylori* T4SS interacts with the host cell either directly, e.g., via integrins, or via translocation of the virulence factor CagA, resulting in phosphorylation of Src followed by phosphorylation of CagA. **(B)** Three different experimental set-ups each consisting of three different SILAC labeling conditions were used. For each set-up, four biological replicates were analyzed. All experiments included non-infected cells for normalization. Experiment 1 was used to analyze time-dependent changes at 90 min and 7 h after infection with wild-type P12. Experiments 2 and 3 were used to analyze CagA- and T4SS-dependent changes 90 min after infection. **(C)** Venn diagram showing the overlap in the different conditions of all 164 phosphopeptides identified and quantified.

The human pathogen *H. pylori* infects nearly 50% of the world's population (Peek and Blaser, 2002). Chronic infection with *H. pylori* may lead to gastritis and gastric or duodenal ulcers and ultimately to the development of gastric cancer or B-cell mucosa-associated lymphoid tissue lymphoma (MALT) (Suerbaum and Michetti, 2002). The most severe sequels of *H. pylori* infection are linked to the presence of a 37 kb Cag pathogenicity island (PAI), which contains up to 31 putative genes, encoding the structural components and effector proteins of a specialized T4SS (Akopyants et al., 1998). The T4SS together with its associated CagL protein has been suggested to interact directly with host cell surface receptors to induce integrin-dependent signaling followed by activation of the focal adhesion kinase (FAK) and subsequent c-SRC phosphorylation, independently of the effector protein CagA (Kwok et al., 2007). Upon binding, the T4SS translocates CagA, which is tyrosine phosphorylated within the host cell by several kinases (Figure 1A), mainly of the SRC and Abl family (Selbach et al., 2002; Stein et al., 2002; Backert and Naumann, 2010). In turn, the CagA-dependent activation of c-terminal SRC kinase (CSK) induces a negative feedback loop leading to the inactivation of SRC and its signaling targets, e.g., cortactin (Tsutsumi et al., 2003).

Phosphorylation of CagA and the subsequent signaling cascade represent key steps in the early phase of infection (Backert and Naumann, 2010). They trigger a tightly controlled sequence of kinase activation and inactivation events, through which CagA interferes with multiple cellular signaling pathways, and thereby controls pro-inflammatory and anti-apoptotic responses, involving NF-κ B- and AP-1-controlled genes, as well as innate defense pathways (Bauer et al., 2012) and cytoskeletal rearrangements (Hatakeyama, 2008; Backert et al., 2010).

In an earlier investigation of *H. pylori*-infected AGS cells, for which phosphothreonine- and phosphoserine-containing protein species were enriched by immobilized metal ion affinity chromatography (IMAC) and analyzed by SILAC-2-DE-MS, we detected protein species-specific regulation of a series of proteins involved in splicing processes (Holland et al., 2011). To gain further insight into the host signaling pathways triggered by *H. pylori* infection, we now used a SILAC-LC-MS approach to analyze Tyr phosphorylation, a hallmark of *H. pylori*-induced signaling (Tegtmeyer and Backert, 2011). Serine and threonine phosphorylation occurs more frequently than tyrosine phosphorylation, generally leading to identification of few phospho-Tyr sites in global phosphorylation screens. However, identification of tyrosine phosphorylation events is of particular interest since they play a major role in transmembrane signal transduction mediated by receptor tyrosine kinases. Therefore, this study was focused on *H. pylori*-induced changes in tyrosine phosphorylation.

A convenient approach to detect the non-abundant Tyr-phosphorylated proteins is to use specific antibodies for phospho-Tyr peptide enrichment followed by sensitive liquid chromatography mass spectrometry (LC-MS) identification (Rush et al., 2005; Rikova et al., 2007; Kruger et al., 2008). Here we applied this procedure to detect changes in protein tyrosine phosphorylation sites upon *H. pylori* infection of AGS cells. We compared different time points and mutant strains to characterize the temporal dynamics of tyrosine phosphorylation changes as well as the impact of relevant bacterial determinants. Our study suggests distinct pathways of phosphotyrosine regulation upon infection. While CagA mainly induced phosphorylation of ERK1 and integrin-linked factors, such as BCAR1, the remaining T4SS components induced phosphorylation predominantly of JNK2/3 and p38.

## Materials and methods

### Cell culture and infection

AGS cells (gastric adenoma carcinoma epithelium cell line CRL 1739, ATCC) were grown in RPMI medium (PAA, Pasching, Austria) without arginine and lysine, supplemented with 10% fetal calf serum (FCS, Sigma Aldrich, Taufkirchen, Germany), 300 mg/L glutamine, and 25 mM HEPES in 75 cm^2^ cell culture flasks at 37°C in a 5% CO_2_ incubator (Holland et al., 2011). Light labeling was obtained by L-arginine (^12^C^14^_6_N_4_) and L-lysine (^12^C^14^_6_N_2_) (both Sigma Aldrich), medium labeling by L-arginine (^13^C^14^_6_N_4_) and D4-L-lysine (^12^C^14^_6_N_2_), heavy labeling by L-arginine (^13^C^15^_6_N_4_), and L-lysine (^13^C^15^_6_N_2_) (all from Silantes, Munich, Germany), resulting in mass shifts of 0/0, 6/4, and 10/8, respectively, as described by Ong and Mann (2006). A labeling rate of 97% was reached after five passages. About 2 × 10^8^ cells per labeling condition were serum starved 20 h prior to infection.

*H. pylori* strains P12 WT, P12 Δ CagA and P12 Δ PAI (Wessler et al., 2000) were inoculated in BHI medium containing 10% heat-inactivated FCS, 0.001% Vancomycin and 1.08 μM Nystatin at an OD550 of 0.2. Cultures were incubated overnight at 37°C, 120 rpm in microaerophilic conditions using jars containing anaerobic sachets (Oxoid, Cambridge, England) to obtain an OD550 of 0.5–0.6. For infection, the bacterial culture was centrifuged (3000 × g for 10 min) and washed twice with phosphate buffered saline (PBS). After the final wash, cells were re-suspended in PBS and used at multiplicity of infection (MOI) 100.

### Sample preparation and tyrosine-phosphopeptide enrichment

After infection for 90 min or 7 h the medium was removed and cells briefly washed with PBS and lysed with 10 ml lysis buffer (20 mM HEPES pH 8.0, 9 M urea, 25 mM sodium pyrophosphate, 10 mM beta-glycerophosphate, and 1 mM sodium vanadate). The lysates from the three labeling states (light, medium, heavy) were combined, resulting in 30 ml (equal to 6 × 10^8^ cells) and sonicated using a Branson Sonifier (step 5, 3 × 30 s, each separated by 1 min on ice). The supernatant of a 20 min, 20,000 × g centrifugation at 4°C was reduced by addition of 3 ml 45 mM DTT and 20 min incubation at 60°C in a water bath. After reduction, the peptides were alkylated with 3 ml 100 mM iodoacetamide and incubation at RT for 15 min in the dark. The alkylated peptides were digested by trypsin in a total volume of 120 ml by addition of TPCK trypsin (Pierce, Rockford, USA) and incubation at RT for 20 h. For enrichment of the peptides, 40 ml of the peptide solution was applied to a SepPak C18 column (Millipore, Bellerica, USA). The column was washed with 12 ml 0.1% trifluoroacetic acid (TFA) followed by 3 ml 5% acetonitrile (ACN), 0.1% TFA. The peptides were eluted with a 5% step gradient from 10 to 40% ACN in 0.1% TFA with 1.5 ml per step. The resulting peptide solution was lyophilized for 2 days.

The lyophilized peptides were solubilized in 4.2 ml of immuno-precipitation buffer containing 50 mM MOPS pH 7.2, 10 mM sodium phosphate, and 50 mM sodium chloride under shaking for 30 min. To remove insoluble material the solution was centrifuged at 2000 × g for 5 min and only the supernatant was used for immuno-precipitation (Rush et al., 2005). Eighty microliter of the immobilized phosphotyrosine-specific antibody (pY-100 for use in Phosphoscan, Cell Signaling, Danvers USA) were incubated with 1.4 ml of the peptide solution rotating over night at 4°C. The antibodies with the bound phosphotyrosines were pelleted by centrifugation at 1500 × g for 1 min at 4°C, the pellet washed six times with 1 ml immuno-precipitation buffer followed by two washes with deionized water. The final pellet containing the phosphotyrosine peptides bound to the antibodies was incubated for 10 min with 100 μl 0.15% TFA to elute the peptides from the antibodies. After a further centrifugation step the supernatant was collected to obtain 100 μl of the phosphotyrosine peptides for each of the three samples. To purify and enrich the peptides, 10 × 10 μl of the peptide solution was bound to a 0.1% TFA, 40% ACN activated and 0.1% TFA washed ZipTip (Millipore) washed twice with 0.1% TFA and eluted with 10 μl 0.1% TFA and 40% ACN. This peptide solution was dried using a SpeedVac concentrator. For each of the experiments four biological replicates were carried out.

### LC-MS analysis

The dried peptides were dissolved in 10 μl formic acid, 5 μl of which were used for LC-MS analysis. To analyze the phosphotyrosine peptides, we used an LC/MS system consisting of a Dionex Ultimate 3000 nano-LC system (Sunnyvale, USA) connected to a linear quadrupole ion trap Orbitrap (LTQ Orbitrap XL) mass spectrometer (ThermoElectron, Bremen, Germany) equipped with a nanoelectrospray ion source. For LC separation, an Acclaim PepMap 100 column (C18, 3 μm, 100 Å) (Dionex) capillary with a 12 cm bed length was used with a flow rate of 300 nl/min. Two solvents, A (0.1% formic acid) and B (aqueous 90% acetonitrile in 0.1% formic acid), were used to elute the tryptic peptides from the nanocolumn. The gradient from 7 to 40% B in 87 min and from 40 to 50% B in 3 min had a total run time of 110 min. The mass spectrometer was operated in the data-dependent mode to automatically switch between Orbitrap-MS and LTQ-MS/MS acquisition. Survey full scan MS spectra (from m/z 300 to 2000) were acquired in the Orbitrap with a resolution *r* = 60,000 at m/z 400 and allowed the sequential isolation of the top six ions, depending on signal intensity. For accurate mass measurements, the lock mass option was enabled in MS mode, and the polydimethylcyclosiloxane ions generated in the electrospray process from ambient air were used for internal recalibration during the analysis (Olsen et al., 2005). Other instrument parameters were set as described elsewhere (Koehler et al., 2009; Thiede et al., 2013).

### Protein identification and quantification

Protein identification and quantification of phosphopeptides were performed with MaxQuant (v.1.3.0.5) (Cox and Mann, 2008) utilizing the Andromeda search engine (Cox et al., 2011) with the IPI human database (v.3.68 – 87.061 human sequences). Trypsin was selected as enzyme allowing two missed cleavage sites, and tolerance levels for identification were set to 10 ppm and 0.5 Da for MS and fragment MS/MS scans, respectively. In addition to stable isotopes of arginine (^13^C_6_^14^N_4_ and ^13^C^15^_6_N_4_) and lysine (^2^H_4_ and ^13^C^15^_6_N_2_), carbamidomethylation of cysteines as fixed modification, and methionine oxidation, phosphorylation of serines, threonines and tyrosines, N-terminal protein acetylation and conversion of N-terminal peptide glutamine to pyro-glutamic acid were selected as variable modifications. We also included the reversed sequences as well as common contaminants into the database search, enabling estimation of the false discovery rate (FDR) which was fixed at 1% for protein and peptide identifications. For quantifications, at least two quantification events were required per phosphopeptide. All evidences with quantification of phosphorylated peptides were used for further analysis. The mass spectrometry proteomics data have been deposited to the ProteomeXchange Consortium (http://proteomecentral.proteomexchange.org) via the PRIDE partner repository (Barsnes et al., 2009) with the dataset identifier PXD000722.

### Functional data analysis

The Uniprot database was used to gain general information (e.g., known phosphorylation sites and other modifications).

Information about regulated phosphopeptides was reduced to the protein level and searched against the Homo Kinase data base (http://www.biomining-bu.in/homokinase/) (Subramani et al., 2013). Kinases were grouped on the level of kinase families. All kinases that were annotated on this level were considered for analysis.

Predicted kinases for regulated phosphopeptides were identified using the NetworKin 2.0 algorithm (Linding et al., 2007). Phosphosites were submitted using Uniprot identifiers. If more than one kinase was predicted to regulate a specific phosphosite, the one with the highest score was chosen and all predicted kinases were grouped at the kinase sub-family level.

## Results

Multiplexed stable-isotope labeling by amino acids (SILAC) was employed to characterize *H. pylori*-induced tyrosine phosphosignaling events upon infection in cell culture (Figure 1B). Multiple sets of isotope-labeled cells were infected for 90 min or 7 h with wild type *H. pylori*, or for 90 min with mutant strains in which either the whole T4SS or only the *cagA* gene was deleted. The resulting data set enabled analysis of temporal aspects of wild type infection and of specific effects of CagA and T4SS on short-term infection. Non-infected cells labeled with the light amino acids Arg(0) and Lys(0) were taken as reference for differential approaches with multiplexed SILAC samples. Infected samples were labeled with either Arg(6)/Lys(4) (medium label) or Arg(10)/Lys(8) (heavy label) and tested for temporal changes and differences in the phosphorylation pattern depending on the presence or absence of CagA or the T4SS (Figure 1B).

Samples were subjected to phosphopeptide enrichment after infection and peptides were quantified using nano-LC-Orbitrap MS. In total, 164 phosphopeptides were identified (Table S1 and Supplementary Data S1) and quantified, 86 of which were present in all conditions (Figure 1C). All phosphosites were annotated with their respective sequences and validated using Uniprot (UniProt) or Phosphosite (PhosphoSitePlus). Phosphosites were classified as regulated if the absolute log2 SILAC ratio of phosphorylation compared to the non-infected control was >0.5 in at least one of the two infection conditions. All regulated phosphopeptides (*n* = 85) were included in the global analysis.

### Kinase family analysis

**Figure 2:**
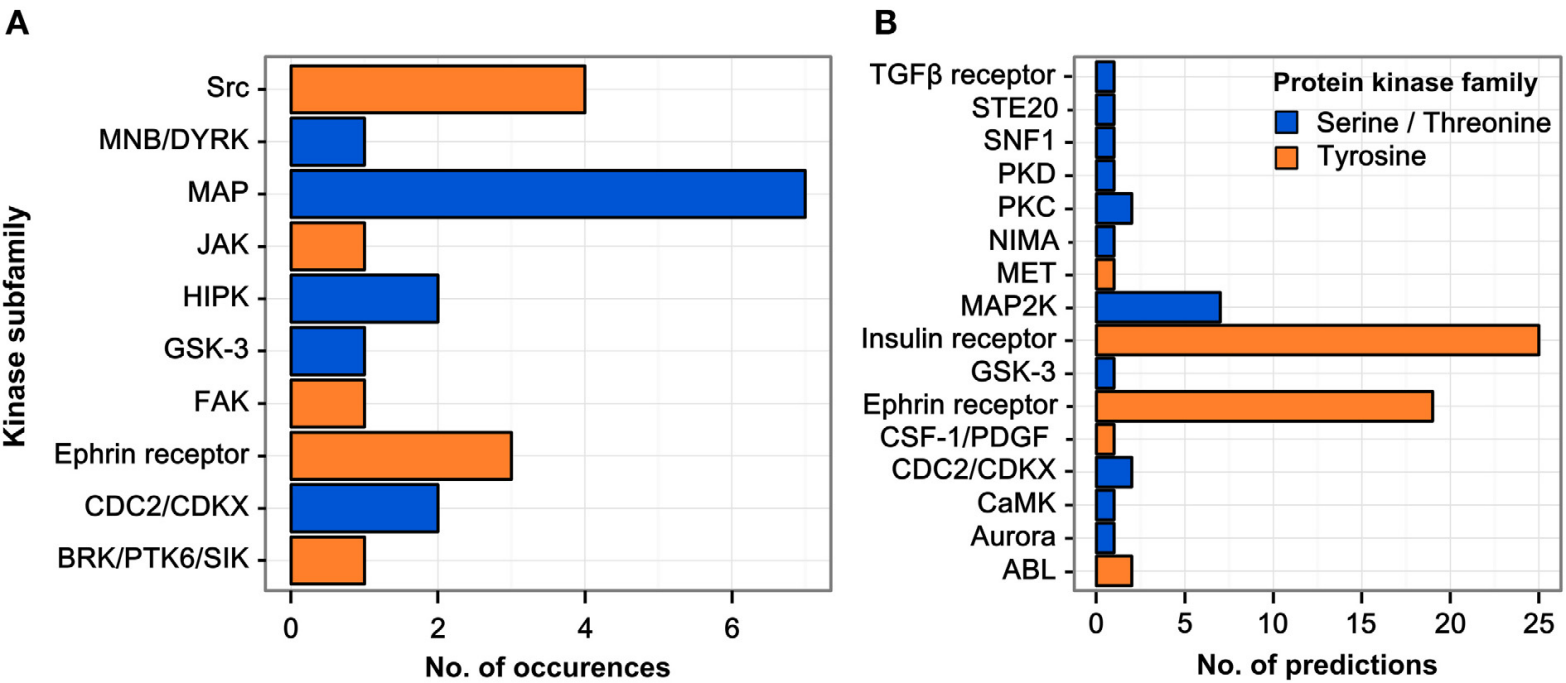
**Regulated kinase families identified and predicted acting/upstream kinase families responsible. (A)** Kinases which were identified among the regulated phosphopeptides were sorted according to their respective kinase subfamily. Only hits with a log2 SILAC ratio > ±0.5 were included. MAP kinases are the most abundant kinase subfamily represented within the dataset. **(B)** All phosphopeptides showing increased phosphorylation upon infection in any condition were scanned for potential kinase target sites using the NetworKIN database. The receptor tyrosine kinase subfamilies of the insulin and ephrin receptors are predicted to target most of the phosphosites regulated during infection.

The parent proteins of regulated phosphopeptides were annotated according to kinase family using the HomoKinase database (http://www.biomining-bu.in/homokinase/) (Subramani et al., 2013). MAP kinases were the most prominently represented kinases (Figure 2A) in congruence with previous findings showing that the ERK/MAPK pathway is one of the key signaling pathways stimulated by *H. pylori* infection (Mitsuno et al., 2001). The color-coding in Figure 2A indicates that several of the kinases phosphorylated at tyrosine residues are serine/threonine-specific, thus providing a link between tyrosine phosphorylation and the serine/threonine phospho-proteome (Holland et al., 2011).

### Kinase target scan

The sequence motifs surrounding the phosphorylated tyrosine residues could provide valuable information on the responsible kinases with their known target site specificities. We therefore matched the 85 regulated phosphosites with potential kinase target sites using the NetworKIN database (Linding et al., 2007) (see Table S2). For each phosphosite the highest-scoring predicted kinase was chosen and grouped according to kinase family (Figure 2B). Two kinase families were found to account for the majority of infection-induced phosphorylation events, i.e., insulin receptor- and ephrin receptor-linked kinases.

### Temporal resolution of phosphorylation events

**Figure 3:**
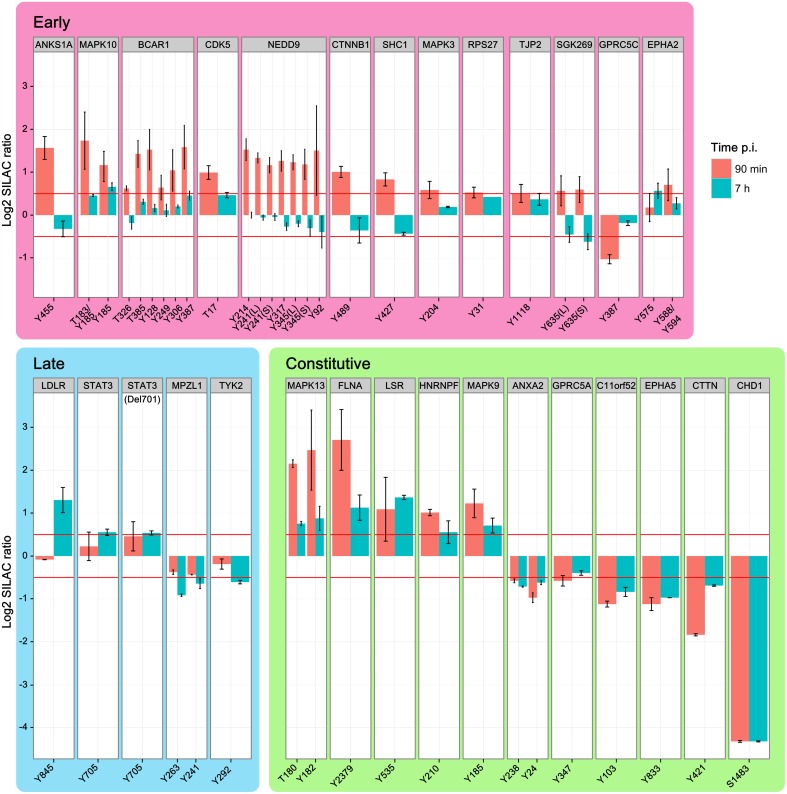
**Temporal regulation of phosphopeptides**. Time-course of phosphopeptide regulation upon infection with *H. pylori* wild type. Shown are the phosphosites and parent proteins, which are significantly regulated at either 90 min or 7 h after infection. Identical phosphosites that were identified in different peptides are differentiated as longer (L) or shorter (S) peptide. While increased phosphorylation was prominent in the early phase of infection the late phase of infection shows mainly decreased phosphorylation compared to non-infected cells. MAPK kinases and integrin-linked factors in particular show strong activation during the early phase of infection, but return to normal levels 7 h after infection. (Data represented as median ± SD).

Although *H. pylori* establishes a long-term infection *in vivo*, individual cells show a clearly orchestrated short-term infection dynamic *in vitro*. Response to interaction with the T4SS starts at ~30 min after infection to initiate subsequent phosphorylation cascades. In total, 28 proteins contained phosphopeptides that were regulated in wild type-infected cells for at least one time point. Phosphosites were grouped into early (13) and late (4) regulated proteins or proteins regulated at both time points (11) (Figure 3). Most phosphosites were regulated within 90 min and returned to the unregulated state by 7 h post-infection. Nineteen proteins showed increased and only 9 proteins showed decreased phosphorylation upon infection.

The early-regulated phosphopeptides consisted of MAPK3 (ERK1), MAPK10 (JNK3), and integrin-regulated proteins. The integrin signaling mediators BCAR1 and NEDD9, as well as MAPK3 and the SRC homology domain-containing protein SHC1, showed a strong, time-dependent phosphorylation after 90 min of infection, returning to control levels by 7 h post-infection. TJP2 was also positively regulated, while GPRC5C was negatively regulated 90 min post-infection. Except for MAPK10 Y185 all phosphosites in this group returned to normal levels by 7 h post-infection. Of the late-regulated phosphopeptides only STAT3, the STAT3 deletion variant Del701, and the low density lipoprotein receptor LDLR showed increased phosphorylation at 7 h post-infection. The remaining proteins in this group—TYK2 and MPZL1—showed decreased phosphorylation. Proteins that were regulated at both timepoints (“constant”) included HNRNPF, FLNA, and MAPK9 (JNK2), which showed increased phosphorylation and C11orf52, CDCP1, CHD1, and cortactin CTTN, which showed decreased phosphorylation.

We also observed phosphosite-specific regulation of EPHA2. At 90 min post-infection, the EPHA2 Y575 phosphosite was not regulated but the Y588/Y594 phosphosite was. After 7 h the Y575 phosphosite was upregulated while the Y588/Y594 phosphosite had returned to control levels. In BCAR1 all phosphotyrosine-containing peptides are upregulated at 90 min, but to a different degree, and all of them are not regulated at 7 h. In conclusion, the temporal regulation reveals a high number of phosphorylation events early upon infection many of which had returned to baseline levels by 7 h.

### Impact of the T4SS

**Figure 4:**
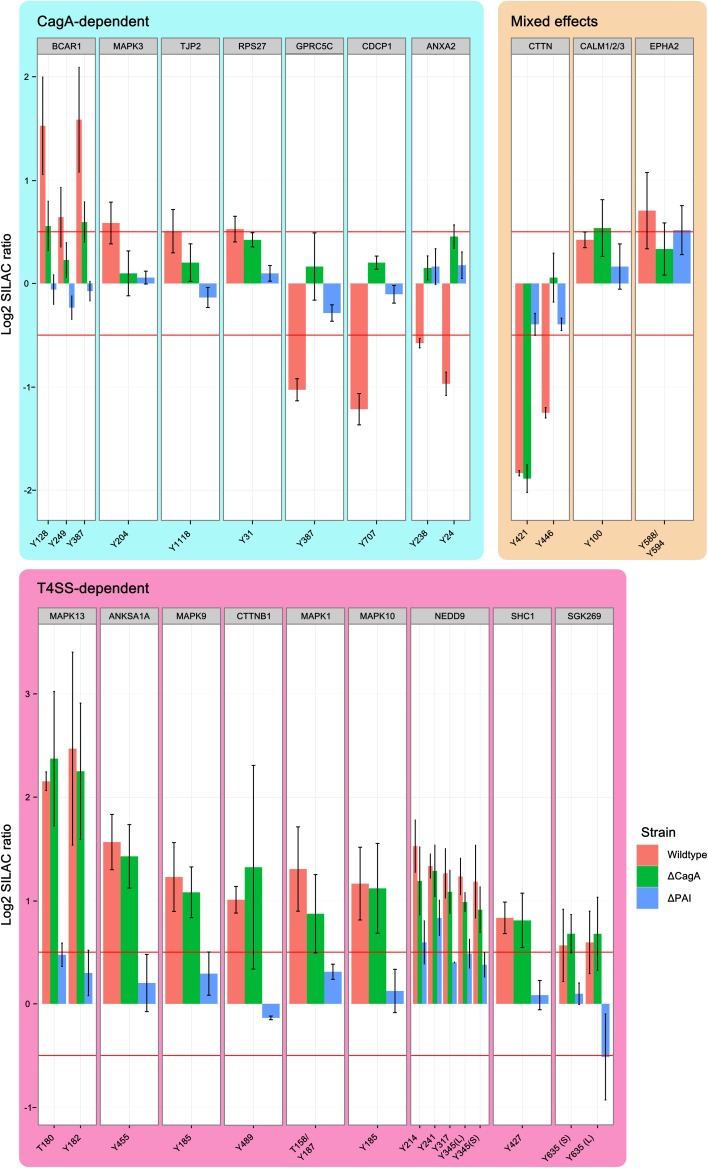
**Effect of the Cag PAI on protein phosphorylation**. Phosphosites regulated by T4SS components 90 min after infection with *H. pylori*. Shown are all phosphopeptides which are regulated in at least one condition, grouped according to whether regulation is dependent on CagA or T4SS. Identical phosphosites that were identified in different peptides are differentiated as longer (L) or shorter (S) peptide. While CagA-regulated phosphosites include examples of both phosphorylation and dephosphorylation in wild-type infected cells, all T4SS-regulated phosphosites show enhanced phosphorylation in all conditions. MAP kinases are strongly represented among the T4SS-regulated phosphosites. (Data represented as median ± SD).

The T4SS is associated with enhanced pathogenicity and contains the secreted bacterial effector protein CagA, as well as the remaining structural components of the secretion machinery. Binding of CagL to integrins on the host cell surface is thought to activate SRC kinases, which in turn phosphorylate translocated CagA on its EPIYA motifs. By using mutant strains of P12 deficient for CagA or the entire T4SS, we were able to distinguish effects that depend on the translocation of CagA or contact of the remaining T4SS with the host cell. Twenty-seven phosphotyrosine sites from 19 proteins were found to be regulated in at least one condition (wild-type, Δ CagA or Δ PAI) at 90 min post-infection and the parent proteins grouped according to their regulatory profile (Figure 4). These common regulatory profiles were used to cluster proteins according to their most likely way of regulation (Figure S1).

### CagA-dependent effects

Phosphosites that were regulated only after infection with wild type P12 but not P12Δ CagA or P12Δ PAI were classified as CagA-dependent. Ten phosphosites of seven proteins were found to be regulated by CagA (“CagA-dependent” in Figure 4). Three proteins, CDCP1, GPRC5C and ANXA2, showed decreased phosphorylation in wild-type- but not P12Δ CagA-infected cells. This suggests CagA has an indirect effect on these phosphosites via activation of cellular phosphatases, such as SHP2, as described by Higashi et al. (2002). A further example of phosphosite-specific regulation was observed for BCAR1. Without CagA, only Y249 but not Y128 or Y387 showed significant phosphorylation, thus rendering these sites T4SS-dependent. In the same group, dephosphorylation of cortactin Y446 was dependent on CagA. Nevertheless, cortactin was grouped as a mixed-effect, since the second phosphosite Y421 was regulated in a T4SS-dependent manner.

### T4SS-dependent effects

Apart from mediating the translocation and phosphorylation of CagA, the T4SS also interacts directly with the host cell surface. By comparing the two deletion mutants, effects induced by the T4SS itself can be distinguished from effects induced by the general interaction of *H. pylori* with the host cell surface in the complete absence of the T4SS. Tyr-phosphosites of 9 proteins were found to be regulated in a T4SS-dependent manner without being specifically dependent on CagA (“T4SS-dependent” in Figure 4). SHC1, NEDD9, and the MAP kinases ERK2 (MAPK1), JNK2 (MAPK9), JNK3 (MAPK 10), and p38 (MAPK13) showed increased phosphorylation whereas cortactin Y241 showed decreased phosphorylation in dependence on the remaining components of the T4SS (Selbach et al., 2003).

### CagPAI-independent effects

Two proteins, calmodulin and EPHA2, were found to be regulated independently of the T4SS (“Mixed effects” in Figure 4). While deletion of CagA abolished the infection-induced increase in EPHA2 phosphorylation, deletion of the T4SS led to an increase in phosphorylation above the cut-off, reminiscent of a compensatory effect of CagA on the effect of the T4SS alone. The phenotype of calmodulin was the reverse of EPHA2.

## Discussion

Host cell protein phosphorylation is one of the evident signaling routes employed during *H. pylori* infection. Here we provide a snapshot of phosphorylation changes in AGS cells. By enriching for tyrosine phosphosites we focused here on a highly dynamic cellular signaling network. We found that phosphorylation changes occurred mainly during the early phase of infection, while few phosphosites were regulated at 7 h post-infection. Interestingly, a large part of the CagA-dependent changes comprised dephosphorylation rather than phosphorylation events. Further, most of the dephosphorylations were stable over time. This suggests either inhibition of cellular kinases or activation of phosphatases, such as SHP-2.

### CagA-dependent effects

CagA-dependent inhibition of SRC was previously shown to be responsible for the tyrosine dephosphorylation of the SRC substrate cortactin (Selbach et al., 2003; Tegtmeyer et al., 2011). Initial activation of SRC and subsequent phosphorylation of the respective tyrosine residues in cortactin (Y421/Y466/Y482) inhibits the interaction of cortactin and the FAK. However, our data suggest a more complex regulatory mechanism for cortactin phosphorylation dynamics. We also observed that cortactin Y446 is dephosphorylated in the presence of CagA. This could be due to inhibition of cellular kinases responsible for cortactin phosphorylation, e.g., SRC. In contrast, cortactin Y421 dephosphorylation was not dependent on CagA. This indicates differential targeting of tyrosine residues of the same protein by phosphatases and kinases.

This observation validates our approach with its focus on Tyr-phosphorylated proteins. Since quantification is reduced to peptides it provides no resolution of protein species, which are the functional units of the proteome (Jungblut et al., 2008). However, the greater sensitivity of detecting phosphorylation sites is highly informative regarding their individual and perhaps differential regulation, as demonstrated here for BCAR1 and EPHA2. These observations also highlight the intricate complexity of phosphorylation signals in proteins, where independently regulated phosphorylation sites exist.

We were also able to resolve a CagA-dependent phosphorylation of ERK1. This contrasts with previous findings suggesting peptidoglycans and subsequent NOD1 signaling as upstream activators of ERK signaling (Viala et al., 2004; Brandt et al., 2005) or a cagPAI-independent EGFR-mediated phosphorylation of ERK1/2 previously reported in AGS cells (Du et al., 2007).

### T4SS-dependent effects

Here we report that the activating phosphorylation of MAPK10, MAPK13, and MAPK9 strongly depends on the presence of the T4SS, consistent with previous work suggesting that TAK1 and JNKs are regulated independently of CagA (Snider et al., 2008; Sokolova et al., 2014). Interaction of *H. plyori* with β1-integrin directly activates JNKs without the involvement of other upstream signaling nodes such as Nod1, Cdc42, Rac1, MKK4, and MKK7. Taken together, this strongly supports a CagA-independent but T4SS-dependent mechanism for the activation of cellular kinases. It might suggest two independent interaction steps between *H. pylori* and β1-integrin: one involving an as yet unknown interactor on the bacterial surface leading to a pre-activation of JNKs and a second involving a strong interaction between the integrin and CagA effector protein, leading to CagA translocation and subsequent signaling (Kaplan-Türköz et al., 2012).

### Phosphosite-specific regulation

A total of 27 phosphosites on the 19 infection-regulated parent proteins were quantified to compare different infection conditions involving wild-type and deletion mutants of *H. pylori* (Figure 4). From four proteins we obtained quantification results for more than one phosphosite. For cortactin clear phosphosite-specific regulation was observed. A search in PhosphoSite Plus (http://www.phosphosite.org) for Tyr-phosphorylation sites of catenin–β isoform1 (CTNNB1) revealed 11 phospho-Tyr sites only for this protein. In our experiments we found only one phosphorylation site. For the time-dependent regulation of EPHA2 we could show that the regulated tyrosine during early infection is the phosphorylated Y588/Y594 position, while Y575 is only regulated later during infection.

Considering these data, it appears that phosphosite-specific regulation is likely to be underestimated in our study and it hence might be worthwhile to further improve the sensitivity. Furthermore, following the notion of a protein code (Sims and Reinberg, 2008), the bottom-up approaches emphasized in this study might need to be complemented by top-down investigations, in which the protein species are separated before they are analyzed.

### Inferring upstream signaling networks

The prediction of the upstream kinases based on target site features provides a valuable approach to infer upstream signaling networks. Here, we identified kinases putatively responsible for enhanced phosphorylation of specific phosphosites. Our predictions extend the *H. pylori*-induced signaling networks beyond the directly generated experimental data.

Ephrin receptor kinases were predicted to act as regulating kinases for a large proportion of the phosphosites found to be regulated during infection. Activation of Eph kinases was previously described in fibroblasts to trigger a signaling cascade, ultimately resulting in changes of actin organization and cell adhesion (Carter et al., 2002). These changes were shown to be dependent on phosphorylation of EPHA2, FAK, BCAR1, and paxilin. Since we observed phosphorylation changes of EPHA2, BCAR1, and paxilin also within our experimental data set (for paxilin see Table S1), Eph kinases are promising candidates for upstream kinases and cell-cell signaling activated during *H. pylori* infection. Since other reports suggest that Eph kinases interfere with integrin-mediated signaling by recruiting SHP-2 directly and dephosphorylating FAK and paxilin (Miao et al., 2000), this connection calls for further investigation. Apart from FAK and SHP-2, which are both targets of *H. pylori*-induced signaling, other important *H. pylori* targets, such as JAK/STAT, might be triggered indirectly. Since all of the proteins we found to be phosphorylated in a CagA-dependent manner were predicted as ephrin receptor kinase targets, this receptor activation is likely to involve the direct or indirect interaction with translocated CagA.

## Conclusion

The data presented here provide a first assessment of tyrosine protein phosphorylation in *H. pylori*-infected cells using a direct and quantitative mass spectrometric approach combined with functional characterization of mutant strains. Interestingly, separate pathways were found to be activated depending on the presence or absence of the T4SS or its effector protein CagA. The distinction of CagA-dependent ERK1 signaling and the T4SS-dependend phosphorylation of JNKs and p38 kinases may help to further differentiate the MAPK signaling routes activated during infection with *H. pylori*. Furthermore, *H. pylori* infection not only led to an increase but also to a decrease of phosphorylation which might indicate the differential activation of both kinases and phosphatases. This study therefore provides a basis for future work with the aim to dissect the regulatory subsets of T4SS-dependent interaction between *H. pylori* and its host cell.

## Author contributions

Carsten Holland and Thomas F. Meyer conceived the study, Carsten Holland and Bernd Thiede performed the experiments and generated the data, Frithjof Glowinski, Carsten Holland, Bernd Thiede, Peter R. Jungblut, and Thomas F. Meyer analyzed and interpreted the data, Frithjof Glowinski, Peter R. Jungblut, and Thomas F. Meyer wrote the manuscript, all authors revised and agreed on the manuscript.

### Conflict of interest statement

The authors declare that the research was conducted in the absence of any commercial or financial relationships that could be construed as a potential conflict of interest.
