# Supplementary material for: Analysis of T4SS-induced signaling by H. pylori using quantitative phosphoproteomics
Source: Front Microbiol. 2014 Jul 18;5:356. doi: 10.3389/fmicb.2014.00356 (PMC4102909; doi:10.3389/fmicb.2014.00356)
Supplement: Supplementary file 1 [file Presentation1.PDF]

SUPPLEMENTARY INFORMATION

Figure S1

Regulation trends used to classify regulated phosphosites in Fig 4.

Table S1

List of all phopshosites identified and quantified in this study. Each phosphosite or combination of phosphosites within a peptide is represented individually. Identical phosphosites that were identified in different peptides are differentiated as longer (L) or shorter (S) peptide. Log2 SILAC ratio represents the median of all quantified phosphopeptides.

Table S2

Results of the kinase prediction using the NetworKIN algorithm. Shown is the raw prediction data summarized in Fig. 2B. All predicted kinases were grouped according to the kinase subfamily and the predicted upstream kinase. If several NetworKIN scores were within 0.01 of each other, several kinases were predicted for a single phosphosite.

Supplementary Data S1

Raw data analysis file showing identification scores and quantification results for each peptide identified. Peptides are shown individually and assigned to the respective experimental condition.

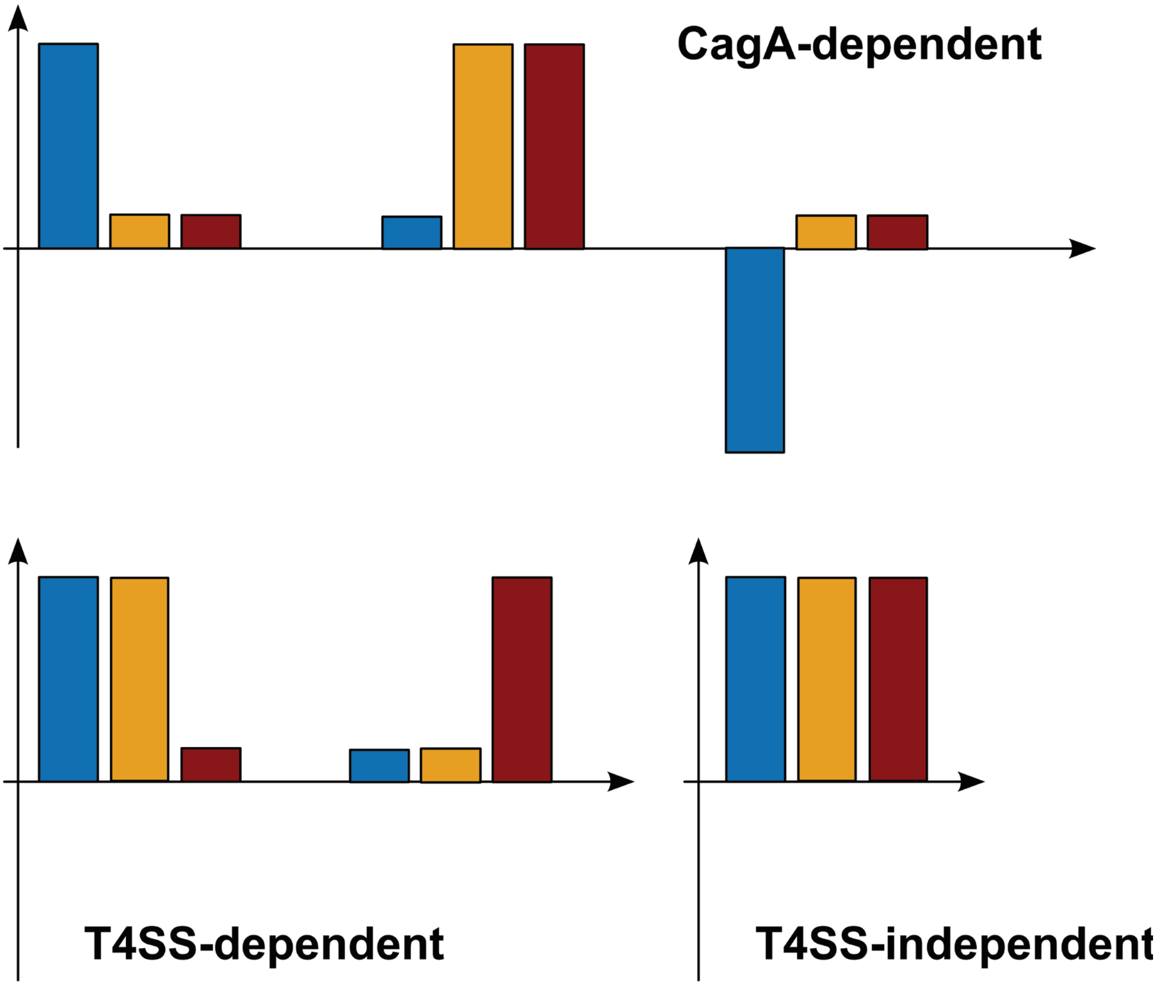

Figure S1

Table S1

Modified.sequence	Phosphosite	HGNC	Uniprot	P12 WT 90 min		P12 dCagA 90 min		P12 dPAI 90 min		P12 WT 7 h	
				Log2 SILAC ratio	Log2 StdDev	Log2 SILAC ratio	Log2 StdDev	Log2 SILAC ratio	Log2 StdDev	Log2 SILAC ratio	Log2 StdDev
TLEPVKPPTVPNDY(ph)MTSPAR	Y213	ABI1	Q8IZP0	0.014	0.003	-0.201	NA	-0.269	NA	0.189	0.132
AIMTYVSSFY(ph)HAFSGAQK	Y265	ACTN4	O43707	-0.105	0.130	-0.184	NA	NA	NA	0.263	0.140
HVSPVT(ph)PPREVPIY(ph)ANR	T761/Y769	ADAM9	Q13443	-0.304	0.294	-0.286	0.155	-0.434	0.063	0.043	NA
IHGSAAREEDEHPY(ph)ELLLTAETK	Y455	ANKS1A	Q92625	1.566	0.268	1.428	0.307	0.202	0.277	-0.322	0.184
AY(ph)TNFDAER	Y30	ANXA2	P07355	0.485	0.240	0.614	0.132	NA	NA	NA	NA
LSLEGDHSTPPS(ph)AYGSVK	S22	ANXA2	P07355	-0.837	0.136	0.516	NA	NA	NA	-0.377	NA
LSLEGDHSTPPSAY(ph)GSVK	Y24	ANXA2	P07355	-0.971	0.113	0.454	0.113	0.176	0.128	-0.621	0.046
LSLEGDHSTPPSAYGS(ph)VK	S26	ANXA2	P07355	-1.089	NA	0.390	0.120	0.070	0.054	NA	NA
SYSPY(ph)DMLESIR	Y238	ANXA2	P07355	-0.578	0.045	0.151	0.116	0.163	0.173	-0.713	0.012
SYSPY(ph)DMLESIRK	Y238	ANXA2	P07355	-0.761	NA	0.151	0.271	0.401	0.224	-0.535	NA
AT(ph)TPPNQGRPDS(ph)PVY(ph)ANLQELK	T230/S240/Y243	ARHGAP12	Q8IWW6	NA	NA	0.189	0.057	-0.252	0.114	NA	NA
GIVVY(ph)TGDR	Y260	ATP1A1	P05023	1.239	0.532	0.506	0.185	NA	NA	NA	NA
AQQGLY(ph)QVPGPSPQFQSPPAK	Y128	BCAR1	P56945	1.526	0.472	0.556	0.240	-0.059	0.142	0.163	0.088
DVPDGPLLREET(ph)YDVPPAFAK	T326	BCAR1	P56945	0.623	0.057	NA	NA	NA	NA	-0.184	0.144
DVPDGPLLREETY(ph)DVPPAFAK	Y327	BCAR1	P56945	0.575	NA	0.299	0.181	-0.074	0.335	0.029	NA
GLPPSNHHAVY(ph)DVPPSVSK	Y306	BCAR1	P56945	1.043	0.484	0.433	0.234	NA	NA	0.202	0.035
HLLAPGPQDIY(ph)DVPPVR	Y249	BCAR1	P56945	0.642	0.288	0.227	0.168	-0.234	0.113	0.111	0.143
RPGPGT(ph)LYDVPR	T385	BCAR1	P56945	1.428	0.313	0.651	NA	NA	NA	0.310	0.060
RPGPGTLY(ph)DVPR	Y387	BCAR1	P56945	1.585	0.507	0.595	0.195	-0.074	0.094	0.454	0.104
HVHLENATEY(ph)ATLR	Y103	C11orf52	Q96A22	-1.120	0.068	0.000	0.142	-0.059	NA	-0.837	0.106
SPGLMSEDSNLHY(ph)ADIQVCSRPHAR	Y78	C11orf52	Q96A22	-1.009	NA	-0.296	NA	-0.219	NA	-1.126	NA
SPGLMSEDSNLHYADIQVCS(ph)RPHAR	S85	C11orf52	Q96A22	-1.143	NA	NA	NA	NA	NA	-1.110	NA
VFDKDGNGY(ph)ISAAELR	Y100	CALM3;CALM2;CALM1	P62158	0.422	0.076	0.536	0.275	0.163	0.219	0.287	0.111
IEKIGEGT(ph)Y(ph)GVVYK	T14/Y15	CDC2	P06493	0.275	0.141	0.444	0.203	0.322	0.116	-0.454	0.097
IEKIGEGTY(ph)GVVYK	Y15	CDC2	P06493	0.287	0.104	0.163	0.093	-0.014	0.130	-0.322	0.086
GPAVGIY(ph)NGNINTEMPR	Y707	CDCP1	Q9H5V8	-1.218	0.151	0.202	0.063	-0.105	0.086	-1.000	NA
IGEGT(ph)Y(ph)GVVYK	T14/Y15	CDK3	Q00526	0.239	0.153	0.433	0.134	0.275	0.042	-0.415	0.028
IGEGTY(ph)GVVYK	Y15	CDK3	Q00526	0.379	0.143	0.356	0.316	0.070	0.055	-0.304	0.100
IGEGTY(ph)GTVFK	Y15	CDK5	Q00535	0.791	NA	0.748	0.674	0.411	0.035	NA	NA
IGEGTYGT(ph)VFK	T17	CDK5	Q00535	0.993	0.159	NA	NA	NA	NA	0.465	0.063
KTPQGPPEIY(ph)SDTQFPSLQSTAK	Y190	CDV3	Q9UKY7	0.263	NA	0.444	NA	-0.218	NA	0.614	NA
HELQANCY(ph)EEVKDR	Y140 (S)	CFL1	P23528	-0.058	0.121	0.158	0.067	NA	NA	NA	NA
LTGIKHELQANCY(ph)EEVKDR	Y140 (L)	CFL1	P23528	-0.047	0.134	0.102	0.057	NA	NA	0.071	0.108
KNLWIFVS(ph)K	S1483	CHD1	O14646	-4.322	0.021	NA	NA	NA	NA	-4.322	0.013
LHY(ph)GLPVVVK	Y489	CTNNB1	P35222	1.007	0.129	1.322	0.986	-0.136	0.018	-0.358	0.293
GPVSGTEPEPVY(ph)SMEAADYR	Y446	CTTN	Q14247	-1.252	0.050	0.057	0.237	-0.396	0.060	NA	NA
LPSSPVY(ph)EDAASFK	Y421	CTTN	Q14247	-1.837	0.026	-1.889	0.133	-0.396	0.106	-0.690	0.016
RDNEVDGQDY(ph)HFVVSR	Y673	DLG3	Q92796	-0.074	0.136	0.151	0.095	0.029	0.046	-0.494	0.058
FIT(ph)LYQLCK	T2025	DNAH9	Q9NYC9	NA	NA	0.756	NA	-3.118	NA	NA	NA
IYQY(ph)IQSR	Y321	DYRK1A	Q13627	-0.029	0.018	0.029	0.076	0.084	0.078	NA	NA
EHALLAY(ph)TLGVK	Y141	EEF1A1	P68104	-0.029	0.070	0.014	0.197	0.202	0.137	0.070	0.044
EHALLAYT(ph)LGVK	T142	EEF1A1	P68104	-0.105	0.004	-0.105	0.264	-0.044	0.108	NA	NA
STTTGHLIY(ph)K	Y29	EEF1A1	P68104	-0.218	0.083	0.057	0.043	NA	NA	NA	NA
(ac)M(ox)HARDFT(ph)VSAMHGDMDQK	Y298	EIF4A1;SNORA67	P60842	-1.434	NA	-1.000	NA	3.323	NA	4.801	NA
QSPEDVY(ph)FSKSEQLKPLK	Y575	EPHA2	P29317	0.176	0.328	0.000	NA	0.000	NA	0.566	0.178
SEQLKPLKT(ph)YVDPHT(ph)YEDPNQAVLK	T587/T593	EPHA2	P29317	0.322	0.274	0.138	0.099	NA	NA	0.239	NA
SEQLKPLKT(ph)YVDPHTY(ph)EDPNQAVLK	T587/Y594	EPHA2	P29317	0.367	0.281	0.227	0.242	0.356	0.177	0.356	NA
SEQLKPLKTY(ph)VDPHT(ph)YEDPNQAVLK	Y588/T593	EPHA2	P29317	0.310	0.063	0.151	0.045	NA	NA	NA	NA
SEQLKPLKTY(ph)VDPHTY(ph)EDPNQAVLK	Y588/594	EPHA2	P29317	0.705	0.369	0.333	0.252	0.516	0.237	0.275	0.130
SEQLKPLKTY(ph)VDPHTYEDPNQAVLK	Y588	EPHA2	P29317	0.333	0.022	0.287	0.259	0.070	0.111	0.411	0.241
TYVDPHT(ph)YEDPNQAVLK	T593	EPHA2	P29317	0.333	0.108	0.239	0.082	NA	NA	0.310	0.116
TYVDPHTY(ph)EDPNQAVLK	Y594	EPHA2	P29317	0.345	0.179	0.263	0.117	-0.120	0.046	0.239	0.228
VLEDDPEATY(ph)TTSGGK	Y772	EPHA2	P29317	-0.396	0.126	0.098	0.145	NA	NA	NA	NA
VLEDDPEATY(ph)TTSGGKIPIR	Y772	EPHA2	P29317	-0.340	0.109	0.138	0.101	0.098	0.011	NA	NA
VLEDDPEATYT(ph)TSGGKIPIR	T773	EPHA2	P29317	0.057	0.068	0.138	NA	0.124	NA	0.239	0.098
VLEDDPEAAY(ph)TTR	Y833	EPHA5	P54756	-1.120	0.151	-0.044	0.185	NA	NA	-0.971	0.001
EAEY(ph)SDKHGQY(ph)LIGHGTK	Y574/Y581	EPHB4	P54760	-0.434	0.197	-0.218	0.154	NA	NA	NA	NA
EAEY(ph)SDKHGQYLIGHGTK	Y574	EPHB4	P54760	-0.556	0.038	-0.494	0.067	NA	NA	NA	NA
RAQIPEGDY(ph)LSYR	Y1104	ERBB2IP	Q96RT1	-0.168	NA	0.124	0.102	0.029	0.025	-0.269	NA
FDTQYPY(ph)GEKQDEFKR	Y66	ERP29	P30040	0.070	0.153	-0.074	0.203	0.000	0.067	-0.201	0.092
KLWLEAMDGKEPIYT(ph)LPAIISK	Y376	FLJ32810	Q96M56	1.183	0.420	0.888	0.480	NA	NA	NA	NA
VHSPSGALEECY(ph)VTEIDQDKYAVR	Y2379	FLNA	P21333	2.708	0.709	2.290	NA	NA	NA	1.129	0.294
HGHY(ph)FVALFDYQAR	Y46	FRK	P42685	0.163	0.114	0.098	0.109	-0.014	0.083	0.251	0.060
AHAWPSPY(ph)KDY(ph)EVKK	Y347/Y350	GPRC5A	Q8NFJ5	-0.535	0.050	0.322	0.280	NA	NA	NA	NA
AHAWPSPY(ph)KDYEVK	Y347	GPRC5A	Q8NFJ5	-0.578	0.122	0.163	0.429	-0.578	NA	-0.396	0.053
NSQVFRNPY(ph)VWD	Y438	GPRC5C	Q9NQ84	NA	NA	-0.168	0.180	-0.415	0.113	NA	NA
VPSEGAY(ph)DIILPR	Y387	GPRC5C	Q9NQ84	-1.029	0.107	0.163	0.325	-0.286	0.079	-0.184	#WERT!
NEEENIY(ph)SVPHDSTQGK	Y1105	GRLF1	Q9NRY4	0.189	0.081	0.356	0.123	NA	NA	NA	NA
SVSSSPWLPQDGFDPSDY(ph)AEPMDAVVKPR	Y1087	GRLF1	Q9NRY4	-0.074	NA	0.057	0.109	-0.089	NA	NA	NA
GEPNVS(ph)YICSR	S278	GSK3A	P49840	-0.040	NA	-0.021	NA	NA	NA	NA	NA
GEPNVSY(ph)ICS(ph)R	Y279/S282	GSK3A	P49840	-0.022	NA	0.275	NA	NA	NA	NA	NA
GEPNVSY(ph)ICSR	Y279	GSK3A	P49840	-0.035	0.029	-0.002	0.093	0.009	0.020	-0.028	0.011
AVCSTY(ph)LQSR	Y352	HIPK1	Q86Z02	-0.116	0.056	-0.007	0.064	NA	NA	NA	NA
TVCST(ph)YLQSR	Y359	HIPK3	Q9H422	-0.347	0.068	0.868	0.009	NA	NA	NA	NA
FMSVQRPGPY(ph)DRPGTAR	Y210	HNRNPF	P52597	1.014	0.074	NA	NA	NA	NA	0.556	0.265
ERLY(ph)EWISIDKDEAGAK	Y886	INPPL1	O15357	-0.136	0.150	0.111	0.062	NA	NA	0.239	0.028
WDTGENPIY(ph)K	Y783	ITGB1	P05556	-0.105	0.089	0.124	0.160	0.084	0.127	0.098	0.157
LIY(ph)LVPEK	Y553	ITSN2	Q9NZM3	NA	NA	0.714	0.037	1.406	0.178	NA	NA
MVAS(ph)M(ox)SKR	S350	KLHL20	Q9Y2M5	NA	NA	0.956	NA	3.632	NA	NA	NA
LIEDNEY(ph)TAR	Y419	LCK	P06239	-1.434	0.008	-0.089	0.051	NA	NA	NA	NA
NLDNGGFY(ph)ISPR	Y192	LCK	P06239	0.333	NA	0.111	0.083	-0.014	0.026	0.189	NA
TTEDEVHICHNQDGY(ph)SYPSR	Y845	LDLR	P01130	-0.083	0.004	NA	NA	NA	NA	1.304	0.294
TTEDEVHICHNQDGYS(ph)YPSR	Y846	LDLR	P01130	-0.003	NA	NA	NA	NA	NA	1.381	NA
SRDDLY(ph)DQDDSRDFPR	Y535	LSR	Q86X29	1.091	0.745	0.299	0.551	0.263	NA	1.367	0.050
VIEDNEY(ph)TAR	Y411	LYN	P08631	-1.059	0.064	0.287	0.068	NA	NA	NA	NA
VADPDHDHTGFLT(ph)EY(ph)VATR	T158/Y187	MAPK1	P28482	1.305	0.408	0.872	0.379	0.310	0.073	0.367	NA

VADPDHDHTGFLTEY(ph)VATR	Y187	MAPK1	P28482	0.422	0.184	0.124	0.125	0.043	0.099	0.098	0.065
TAGTSFMMT(ph)PY(ph)VVTR	T183/Y185	MAPK10	P53779	1.736	0.667	NA	NA	NA	NA	0.454	0.023
TAGTSFMMT(ph)PYVVTR	T183	MAPK10	P53779	NA	NA	1.251	0.153	0.411	0.249	NA	NA
TAGTSFMMTPY(ph)VVTR	Y185	MAPK10	P53779	1.163	0.352	1.118	0.435	0.124	0.210	0.660	0.089
HADAEM(ox)T(ph)GYVVTR	T180	MAPK13	O15264	2.425	0.234	2.157	0.206	NA	NA	NA	NA
HADAEM(ox)TGY(ph)VVTR	Y182	MAPK13	O15264	2.503	0.749	2.070	0.289	NA	NA	NA	NA
HADAEMT(ph)GYVVTR	T180	MAPK13	O15264	2.154	0.090	2.373	0.651	0.475	0.113	0.757	0.051
HADAEMTGY(ph)VVTR	Y182	MAPK13	O15264	2.470	0.934	2.251	0.660	0.299	0.220	0.880	0.280
HTDDEMTGY(ph)VATR	Y182	MAPK14	Q16539	1.655	0.186	1.501	0.220	NA	NA	NA	NA
IADPEHDHTGFLTEY(ph)VATR	Y204	MAPK3	P27361	0.585	0.202	0.098	0.217	0.057	0.062	0.189	0.016
GLCTSPAEHQYFMTEY(ph)VATR	Y221	MAPK7	Q13164	0.355	0.204	-0.077	0.107	-0.067	0.105	-0.448	0.079
TACTNFMMTPY(ph)VVTR	Y185	MAPK9	P45984	1.227	0.333	1.079	0.246	0.292	0.209	0.711	0.174
SESVVY(ph)ADIR	Y263	MPZL1	O95297	-0.434	0.006	-0.234	0.114	-0.184	0.239	-0.644	0.114
SLPSGSHQGPVIY(ph)AQLDHSGGHHSDKINK	Y241	MPZL1	O95297	-0.377	0.053	-0.059	0.066	-0.269	0.016	-0.916	0.028
T(ph)PAPVT(ph)STGSTTATLEGQSTAASSR	T40/T45	MUC4	Q99102	0.043	0.061	0.043	0.013	0.014	NA	0.098	0.009
LQLAM(ox)EM(ox)VGFLPKT(ph)RR	T415	MYO9A	B2RTY4	-0.029	0.056	0.000	0.003	-0.234	NA	-0.136	0.055
ANPQERDGVY(ph)DVPLHNPPDAK	Y345 (L)	NEDD9	Q14511	1.233	0.177	0.986	0.090	0.485	0.139	-0.201	0.070
DEAGLREKDY(ph)DFPPPMR	Y241 (L)	NEDD9	Q14511	1.333	0.116	1.287	0.249	0.832	0.171	-0.059	0.061
DGVY(ph)DVPLHNPPDAK	Y345 (S)	NEDD9	Q14511	1.183	0.353	0.911	0.221	0.379	0.119	-0.304	0.194
EKDY(ph)DFPPPMR	Y241 (S)	NEDD9	Q14511	1.163	0.177	0.956	0.237	NA	NA	-0.044	0.080
GPVFSVPVGEIKPQGVY(ph)DIPPTK	Y214	NEDD9	Q14511	1.526	0.253	1.189	0.329	0.595	0.208	0.000	0.067
LY(ph)QVPNPQAAPR	Y92	NEDD9	Q14511	1.506	1.043	1.239	0.190	NA	NA	-0.396	0.372
RHQSLSPNHPPPQLGQSVGSQNDAY(ph)DVPR	Y317	NEDD9	Q14511	1.263	0.239	1.084	0.209	0.401	0.003	-0.269	0.088
TGHGYVY(ph)EYPSR	Y166	NEDD9	Q14511	2.032	0.511	1.438	0.499	NA	NA	NA	NA
YTEFY(ph)HVPTHSDASK	Y251	PDLIM5	Q96HC4	0.202	0.246	0.189	0.278	NA	NA	NA	NA
SREYDRLY(ph)EEYTR	Y467	PIK3R1	P27986	-0.152	0.075	0.111	0.117	0.299	0.066	-0.089	0.116
NNYALNTT(ph)ATYAEPYRPIQYR	T475	PKP4	Q99569	NA	NA	-0.044	NA	-0.044	NA	NA	NA
NNYALNTTAT(ph)YAEPYRPIQYR	T477	PKP4	Q99569	-0.120	0.103	0.176	0.149	0.214	0.175	-0.340	0.079
NNYALNTTATY(ph)AEPYRPIQYR	Y478	PKP4	Q99569	-0.218	0.155	0.390	0.273	NA	NA	-0.358	0.004
YNTM(ox)LGY(ph)KR	Y106	PLAGL1	Q9UM63	NA	NA	0.872	NA	3.558	NA	NA	NA
LCDFGSASHVADNDIT(ph)PYLVSR	T847	PRPF4B	Q13523	0.152	NA	0.167	NA	0.113	NA	0.060	NA
LCDFGSASHVADNDITPY(ph)LVSR	Y849	PRPF4B	Q13523	0.095	0.078	0.040	0.064	0.042	0.064	0.063	0.044
LCDFGSASHVADNDITPYLVS(ph)R	S852	PRPF4B	Q13523	0.198	NA	0.058	NA	0.024	NA	0.001	NA
YM(ox)EDS(ph)TYYK	S574	PTK2	Q05397	0.214	0.005	0.799	0.396	NA	NA	NA	NA
YM(ox)EDSTY(ph)YK	Y576	PTK2	Q05397	0.227	0.086	0.848	0.262	NA	NA	NA	NA
YMEDSTY(ph)Y(ph)KASK	Y576/Y577	PTK2	Q05397	0.000	0.186	0.757	0.205	NA	NA	NA	NA
YMEDSTY(ph)YK	Y576	PTK2	Q05397	0.239	0.055	0.669	0.041	NA	NA	NA	NA
VSEKPSADY(ph)VLSVR	Y114	PTK6	Q13882	-0.474	0.188	-0.578	NA	NA	NA	-0.340	NA
VVQEYIDAFSDY(ph)ANFK	Y798	PTPRA	P18433	-0.340	0.121	0.014	0.137	-0.120	0.077	-0.340	0.047
KYGLFKEENPY(ph)AR	Y174 (L)	PTTG1IP	P53801	-0.152	0.206	-0.074	NA	NA	NA	0.163	0.235
YGLFKEENPY(ph)AR	Y174 (S)	PTTG1IP	P53801	-0.377	0.164	-0.136	0.052	-0.252	0.064	-0.105	0.179
FIHQQPQSSSPVY(ph)GSSAK	Y88	PXN	P49023	0.678	0.113	0.705	0.078	NA	NA	NA	NA
VGEEEHVY(ph)SFPNK	Y118	PXN	P49023	0.475	0.108	0.422	0.053	NA	NA	-0.358	0.093
VGEEEHVY(ph)SFPNKQK	Y118	PXN	P49023	0.678	0.079	0.465	0.143	NA	NA	NA	NA
VGEEEHVYS(ph)FPNK	S119	PXN	P49023	0.516	0.004	0.356	0.008	NA	NA	NA	NA
VGEEEHVYS(ph)FPNKQK	S119	PXN	P49023	0.731	0.197	0.595	0.306	NA	NA	NA	NA
RPLHPALNQPGGLQPLSFQNPVY(ph)HLNNPIPAMPK	Y786	RASAL2	Q2TB22	-0.014	0.197	-0.269	0.106	-0.322	0.090	-0.136	0.079
IAIY(ph)ELLFK	Y12	RPS10	P46783	0.390	0.160	0.251	0.147	-0.029	0.097	-0.168	0.046
LVQSPNS(ph)YFMDVK	S30	RPS27	P42677	0.390	0.164	0.111	0.179	0.367	NA	0.526	NA
LVQSPNSY(ph)FMDVK	Y31	RPS27	P42677	0.526	0.124	0.422	0.069	0.098	0.076	0.422	0.055
(gl)QVIQT(ph)PLADS(ph)LPVSR	Y198/S203	SBF1	O95248	-0.059	0.071	0.043	0.144	-0.105	0.131	-0.152	0.099
CPPAY(ph)TMVGLHNLEPR	Y160	SgK223	Q86YV5	0.845	NA	0.636	NA	0.372	NA	0.374	NA
NAIKVPIVINPNAY(ph)DNLAIYK	Y635 (L)	SGK269	Q9H792	0.566	0.350	0.678	0.186	0.098	0.104	-0.454	0.181
VPIVINPNAY(ph)DNLAIYK	Y635 (S)	SGK269	Q9H792	0.595	0.302	0.678	0.353	-0.515	0.416	-0.621	0.185
AGKGESAGY(ph)MEPYEAQR	Y268	SHB	Q15464	-0.415	0.122	0.124	0.115	NA	NA	NA	NA
DKVTIADDY(ph)SDPFDAK	Y246	SHB	Q15464	-0.218	0.122	-0.074	0.236	0.299	0.174	-0.014	0.061
DKVTIADDY(ph)SDPFDAKNDLK	Y246	SHB	Q15464	-0.377	0.080	-0.089	0.112	0.057	NA	0.014	0.185
DKVTIADDYS(ph)DPFDAK	S247	SHB	Q15464	-0.358	0.090	0.138	0.104	NA	NA	-0.152	0.049
DKVTIADDYS(ph)DPFDAKNDLK	S247	SHB	Q15464	-0.322	0.183	-0.074	0.205	0.000	0.022	-0.089	0.111
ELFDDPSY(ph)VNVQNLDK	Y427	SHC1	P29353	0.832	0.153	0.807	0.264	0.084	0.142	-0.434	0.034
(ac)M(ox)T(ph)SASPEDQNAPVGCPKGAR	T2	SPATA13	Q96N96	NA	NA	-5.676	NA	-5.205	NA	NA	NA
YCRPESQEHPEADPGAAPY(ph)LK	Y705	STAT3	P40763	0.225	0.332	-0.051	0.197	0.172	0.056	0.555	0.071
YCRPESQEHPEADPGS(ph)AAPYLK	S701	STAT3	P40763	NA	NA	-0.061	NA	0.274	NA	NA	NA
YCRPESQEHPEADPGSAAPY(ph)LK	Y705	STAT3 (Del701)	P40763	0.459	0.341	-0.039	0.116	0.214	0.082	0.538	0.047
HPDIY(ph)AVPIK	Y1118	TJP2	Q9UDY2	0.275	0.142	0.239	0.264	-0.218	0.138	0.411	0.046
IEIAQKHPDIY(ph)AVPIK	Y1118	TJP2	Q9UDY2	0.506	0.209	0.202	0.182	-0.136	0.097	0.367	0.139
ALDY(ph)YMLR	Y70	TLN1	Q9Y490	0.163	0.131	0.111	0.061	-0.029	0.064	0.333	0.101
TMQFEPSTMVY(ph)DACR	Y26	TLN1	Q9Y490	0.216	0.165	0.325	0.144	-0.010	0.185	0.122	0.318
KLS(ph)LGQYDNDAGGQLPFSK	S776	TNS3	Q68CZ2	-0.434	NA	-0.152	NA	0.176	NA	-0.074	NA
KLSLGQY(ph)DNDAGGQLPFSK	Y780	TNS3	Q68CZ2	-0.136	0.052	-0.136	0.074	-0.014	0.090	-0.184	0.058
LLAQAEGEPCY(ph)IR	Y292	TYK2	P29597	-0.188	0.119	-0.039	0.185	-0.108	0.179	-0.609	0.041
Y(ph)LSAGPTLQY(ph)DK	Y444/Y452	VANGL1	Q8TAA9	0.098	NA	0.496	NA	NA	NA	NA	NA
VQIY(ph)HNPTANSFR	Y39	VASP	P50552	-0.322	NA	-0.218	0.080	-0.286	0.189	NA	NA
VIY(ph)DFIEK	Y256	WASL	O00401	-0.415	0.179	0.176	0.268	-0.089	0.163	-0.286	0.120
KLDNGGY(ph)YITTR	Y222	YES1	P07947	-0.136	0.058	-0.120	0.067	NA	NA	NA	NA
LVPTGPT(ph)HREPSPVRY(ph)DNLSR	T524/Y533	ZDHHC5	Q9C0B5	0.287	0.137	-0.105	NA	-0.074	NA	0.111	0.083
LVPTGPTHREPS(ph)PVRY(ph)DNLSR	S529/Y533	ZDHHC5	Q9C0B5	0.163	0.113	0.202	0.112	NA	NA	0.070	NA
LENY(ph)S(ph)NLVSVGY(ph)HVSK	Y43/S44/Y51	ZNF334	Q5XKG8	0.475	0.059	0.687	0.024	NA	NA	NA	NA

Table S2

Kinase subfamily	Predicted Kinase	Substrate	Phosphorylation Site	NetworKIN Score
ABL	ABL1	CTNNB1	Y489	2.314
		TYK2	Y292	1.5857
Aurora	STK6	ANXA2	S26	4.6471
		CHD1	S1483	4.5315
CaMK	CAMK2A	DNAH9	T2025	1.0901
		EIF4A1	T298	6.5954
CDC2/CDKX	CDK2	BCAR1	T385	3.1527
		MAPK10	T221	13.3188
CDC2/CDKX	CDK3	BCAR1	T385	3.064
		MAPK10	T221	12.9444
CDC2/CDKX	ICK	ANXA2	S22	4.5788
CDC2/CDKX	MOK	ANXA2	S22	4.6397
CSF-1/PDGF receptor	CSF1R	STAT3	Y705	1.2409
		TYK2	Y292	1.4594
CSF-1/PDGF receptor	FLT3	STAT3	Y705	1.211
		TYK2	Y292	1.4242
CSF-1/PDGF receptor	KIT	STAT3	Y705	1.2421
		TYK2	Y292	1.4609
DMPK	CDC42BPA	BCAR1	T385	2.3229
		EIF4A1	T298	5.6776
DMPK	DMPK	BCAR1	T385	2.4292
		DNAH9	T2025	1.0189
		EIF4A1	T298	5.9392
EGF receptor	EGFR	TYK2	Y292	1.4114
EGF receptor	ERBB2	TYK2	Y292	1.4114
EGF receptor	ERBB3	TYK2	Y292	1.4072
EGF receptor	ERBB4	TYK2	Y292	1.4043
Ephrin receptor	EPHA3	ANKS1	Y455	2.4704
		BCAR1	Y128	7.5194
		BCAR1	Y249	10.0021
		BCAR1	Y306	8.25
		BCAR1	Y327	7.928
		BCAR1	Y387	7.8348
		EPHA2	Y588	7.2722
		EPHA2	Y594	9.2983
		EPHB4	Y574	6.2003
		GPRC5A	Y350	3.9142
		GPRC5C	Y387	3.336
		HNRPF	Y210	4.8584
		ITSN2	Y553	4.3121
		LDLR	Y845	3.8078
		MAPK1	Y187	3.236
		MAPK3	Y204	3.236
		MPZL1	Y241	4.2039
		NEDD9	Y214	6.9067
		NEDD9	Y241	8.299
		NEDD9	Y317	6.36
		NEDD9	Y345	8.9354
		NEDD9	Y92	9.3303
		PXN	Y118	4.5391
		TJP2	Y1118	7.1248
Ephrin receptor	EPHA4	ANKS1	Y455	2.4858
		BCAR1	Y128	7.5654
		BCAR1	Y249	10.0634
		BCAR1	Y306	8.3005

		BCAR1	Y327	7.9765
		BCAR1	Y387	7.8827
		EPHA2	Y588	7.2868
		EPHA2	Y594	9.317
		EPHB4	Y574	6.238
		GPRC5A	Y350	3.9384
		GPRC5C	Y387	3.3556
		HNRPF	Y210	4.8883
		ITSN2	Y553	4.3396
		LDLR	Y845	3.8312
		MAPK1	Y187	3.2558
		MAPK3	Y204	3.2558
		MPZL1	Y241	4.23
		NEDD9	Y214	6.949
		NEDD9	Y241	8.3498
		NEDD9	Y317	6.3989
		NEDD9	Y345	8.9901
		NEDD9	Y92	9.3875
		PXN	Y118	4.5669
		TJP2	Y1118	7.1687
Ephrin receptor	EPHA5	ANKS1	Y455	2.4601
		BCAR1	Y128	7.4887
		BCAR1	Y249	9.9613
		BCAR1	Y306	8.2163
		BCAR1	Y327	7.8956
		BCAR1	Y387	7.8028
		EPHA2	Y588	7.2284
		EPHA2	Y594	9.2422
		EPHB4	Y574	6.1751
		GPRC5A	Y350	3.8997
		GPRC5C	Y387	3.3242
		HNRPF	Y210	4.8384
		ITSN2	Y553	4.2956
		LDLR	Y845	3.7921
		MAPK1	Y187	3.2228
		MAPK3	Y204	3.2228
		MPZL1	Y241	4.1909
		NEDD9	Y214	6.8785
		NEDD9	Y241	8.2652
		NEDD9	Y317	6.334
		NEDD9	Y345	8.8989
		NEDD9	Y92	9.2923
		PXN	Y118	4.5206
		TJP2	Y1118	7.0956
Ephrin receptor	EPHA6	ANKS1	Y455	2.3831
		BCAR1	Y128	7.2662
		BCAR1	Y249	9.6653
		BCAR1	Y306	7.9722
		BCAR1	Y327	7.661
		BCAR1	Y387	7.5709
		EPHA2	Y588	7.0091
		EPHA2	Y594	8.9619
		EPHB4	Y574	5.9927
		GPRC5A	Y350	3.7791
		GPRC5C	Y387	3.2222
		HNRPF	Y210	4.6938

		ITSN2	Y553	4.1636
		LDLR	Y845	3.6788
		MAPK1	Y187	3.1271
		MAPK3	Y204	3.1271
		NEDD9	Y214	6.6741
		NEDD9	Y241	8.0196
		NEDD9	Y317	6.1458
		NEDD9	Y345	8.6345
		NEDD9	Y92	9.0162
		PXN	Y118	4.3862
		TJP2	Y1118	6.8837
Ephrin receptor	EPHA7	ANKS1	Y455	2.4755
		BCAR1	Y128	7.5347
		BCAR1	Y249	10.0225
		BCAR1	Y306	8.2668
		BCAR1	Y327	7.9441
		BCAR1	Y387	7.8508
		EPHA2	Y588	7.2795
		EPHA2	Y594	9.3076
		EPHB4	Y574	6.2128
		GPRC5A	Y350	3.919
		GPRC5C	Y387	3.3438
		HNRPF	Y210	4.8684
		ITSN2	Y553	4.3231
		LDLR	Y845	3.8156
		MAPK1	Y187	3.2426
		MAPK3	Y204	3.2426
		MPZL1	Y241	4.2126
		NEDD9	Y214	6.9208
		NEDD9	Y241	8.316
		NEDD9	Y317	6.373
		NEDD9	Y345	8.9537
		NEDD9	Y92	9.3494
		PXN	Y118	4.5483
		TJP2	Y1118	7.1395
GSK-3	GSK3A	ANXA2	S22	6.2412
		BCAR1	T385	2.3971
GSK-3	GSK3B	ANXA2	S22	6.2603
		BCAR1	T385	2.3995
Insulin receptor	IGF1R	ANKS1	Y455	3.0897
		ANXA2	Y238	6.1745
		ANXA2	Y24	4.2998
		ANXA2	Y30	6.1181
		ATP1A1	Y260	2.0826
		CALM1	Y100	6.258
		CDCP1	Y707	2.892
		CDK5	Y15	6.2826
		CTTN	Y421	6.1645
		EPHA2	Y575	6.1788
		EPHA5	Y833	6.0983
		EPHB4	Y574	5.8644
		GPRC5A	Y347	5.2298
		GPRC5A	Y350	5.1114
		HCK	Y411	6.1313
		HIPK3	Y359	1.6095
		HNRPF	Y210	5.4135

		MPZL1	Y241	5.9828
		MPZL1	Y263	6.1744
		NEDD9	Y317	6.1696
		PLAGL1	Y106	3.5605
		PTK2	Y576	6.3213
		PTK2	Y577	6.2661
		PTK6	Y114	4.7792
		PXN	Y118	4.9326
		RPS27	Y31	4.0182
		SHC1	Y427	6.19
		TJP2	Y1118	6.2832
Insulin receptor	INSR	ANKS1	Y455	3.0897
		ANXA2	Y238	6.1745
		ANXA2	Y24	4.2998
		ANXA2	Y30	6.1181
		ATP1A1	Y260	2.0826
		CALM1	Y100	6.258
		CDCP1	Y707	2.892
		CDK5	Y15	6.2889
		CTTN	Y421	6.1645
		EPHA2	Y575	6.1788
		EPHA5	Y833	6.0983
		EPHB4	Y574	5.8644
		GPRC5A	Y347	5.2298
		GPRC5A	Y350	5.1114
		HCK	Y411	6.1313
		HIPK3	Y359	1.6095
		HNRPF	Y210	5.4135
		MPZL1	Y241	5.9828
		MPZL1	Y263	6.1744
		NEDD9	Y317	6.1696
		PLAGL1	Y106	3.5605
		PTK2	Y576	6.3213
		PTK2	Y577	6.2661
		PTK6	Y114	4.7841
		PXN	Y118	4.9326
		RPS27	Y31	4.0182
		SHC1	Y427	6.19
		TJP2	Y1118	6.2832
MAP2K	MAP2K3	BCAR1	T385	2.4093
		CTNNB1	Y489	1.9739
		CTTN	Y446	2.1975
		MAPK10	Y223	2.4552
		MAPK13	T180	2.8302
		MAPK13	Y182	2.341
		MAPK14	Y182	2.4876
		MAPK9	Y185	2.4552
		PXN	Y88	2.7903
MAP2K	MAP2K4	BCAR1	T385	2.4141
		CTNNB1	Y489	1.9779
		CTTN	Y446	2.2019
		MAPK10	Y223	2.4601
		MAPK13	T180	2.8274
		MAPK13	Y182	2.3386
		MAPK14	Y182	2.4851
		MAPK9	Y185	2.4626

		PXN	Y88	2.7959
MAP2K	MAP2K6	BCAR1	T385	2.4093
		CTNNB1	Y489	1.9739
		CTTN	Y446	2.1975
		MAPK10	Y223	2.4552
		MAPK13	T180	2.8359
		MAPK13	Y182	2.3457
		MAPK14	Y182	2.4876
		MAPK9	Y185	2.4552
		PXN	Y88	2.7903
MAP2K	MAP2K	BCAR1	T385	2.402
		CTNNB1	Y489	1.9679
		CTTN	Y446	2.1908
		MAPK10	Y223	2.4478
		MAPK13	T180	2.816
		MAPK13	Y182	2.3292
		MAPK14	Y182	2.4727
		MAPK9	Y185	2.4503
		PXN	Y88	2.7819
MET	MET	FLNA	Y2379	2.5304
MET	MST1R	FLNA	Y2379	2.4459
NIMA	NEK2	KLHL20	S350	6.1502
PKC	PRKCA	ANXA2	S26	5.4324
		PXN	S119	3.831
PKC	PRKCB1	ANXA2	S26	5.3822
		PXN	S119	3.7807
PKC	PRKCD	ANXA2	S26	5.4603
		PXN	S119	3.8503
PKC	PRKCE	ANXA2	S26	5.293
		PXN	S119	3.7343
PKC	PRKCG	ANXA2	S26	5.4213
		PXN	S119	3.8232
PKC	PRKCI	ANXA2	S26	5.438
		PXN	S119	3.8348
PKC	PRKCQ	ANXA2	S26	5.4324
		PXN	S119	3.8194
PKC	PRKCZ	ANXA2	S26	5.438
		PXN	S119	3.831
PKD	PRKD1	BCAR1	T326	8.9876
SNF1	PRKAA1	DNAH9	T2025	1.4545
		MAPK13	T180	1.9872
SNF1	PRKAA2	DNAH9	T2025	1.4438
STE20	MAP4K4	CDK5	T17	4.596
STE20	MST2	CDK5	T17	5.8688
STE20	STK24	CDK5	T17	5.744
STE20	STK4	CDK5	T17	5.7838
STE20	TNIK	CDK5	T17	4.5676
TEC	BTK	PXN	Y88	2.4549
TEC	ITK	PXN	Y88	2.4549
TEC	TEC	PXN	Y88	2.4426
TEC	TXK	PXN	Y88	2.3835
TGFB receptor	ACVR2B	PTK2	S574	6.1131
TGFB receptor	TGFBR2	PTK2	S574	6.1628
TGFB receptor		PTK2	S574	6.1317
